# Mesenchymal stem cells protect against malaria pathogenesis by reprogramming erythropoiesis in the bone marrow

**DOI:** 10.1038/s41420-020-00363-2

**Published:** 2020-11-15

**Authors:** Reva S. Thakur, Vikky Awasthi, Anirban Sanyal, Samit Chatterjee, Swati Rani, Rubika Chauhan, Meenu Kalkal, Mrinalini Tiwari, Veena Pande, Jyoti Das

**Affiliations:** 1 Parasite-Host Biology, National Institute of Malaria Research, Dwarka, New Delhi 110077 India; 2 Department of Zoology, University of Rajasthan, Jaipur, 302004 India; 3 Department of Genetics and Epigenetics, Institute of Nuclear Medicine & Allied Science, Timarpur, New Delhi 110054 India; 4 Department of Biotechnology, Kamaun University, Nanital, 263001 India

**Keywords:** Lymphocyte differentiation, Malaria

## Abstract

Malaria remains a major public health problem worldwide. The immune mechanisms that mediate protection against malaria are still unclear. Previously, we reported that mesenchymal stem cells (MSCs) play a critical role in host protection against malaria by altering the dynamic balance of T regulatory cells and effector T cells producing inflammatory cytokines. Here, we report that MSCs reprogram haematopoiesis in primary (bone marrow) and secondary (spleen) lymphoid organs to provide host protection against malaria. Adoptive transfer of MSCs from malaria-infected mice to naïve recipient mice that were subsequently infected with malaria parasites dramatically accelerated the formation of colony-forming units-erythroid cells in the bone marrow. Adoptively transferred MSCs also induced expression of the key erythroid cell differentiation factor GATA-1 in the spleen of recipient animals. Interestingly, we further observed a subtle increase in the CD34^+^ hematopoietic stem and progenitor cells in lymphoid organs, including spleen and lymph nodes. Infusion of MSCs also enhanced T cell proliferation, resulting in increased numbers of both CD4^+^ and CD8^+^ T cells in the spleen. MSCs also inhibited the induction of the negative co-stimulatory receptor programmed death-1 by T cells in recipient animals upon infection with malaria parasites. Taken together, our findings suggest that MSCs play a critical role in host protection against malaria infection by modulating erythropoiesis and lymphopoiesis.

## Introduction

Mesenchymal stromal cells or mesenchymal stem cells (MSCs) are multipotent cells, first described by Friedenstein and colleagues in the 1970s in bone marrow, that exhibit the capacity to adhere to plastic in culture^1^. MSCs have self-renewal and differentiation capacity into tissues of mesodermal origin, and support haematopoiesis through the release of various molecules that play important roles in the migration, homing, self-renewal, proliferation, and differentiation of hematopoietic stem and progenitor cells (HSPCs). MSCs have the capacity to differentiate into various cell types such as osteoblasts^2^, chondrocytes^3^ and adipocytes^4,5^. MSCs have tissue regenerative^6^ activities, as well immunomodulatory^7^ properties, as evidenced by their capacity to inhibit the effector functions of T cells^8^, B cells^9^, natural killer (NK) cells^10^, and dendritic cells (DC)^11^ in vitro. Multiple lines of evidence have revealed that the immunosuppressive properties of MSCs are induced by their microenvironment^12^. Recent reports have indicated that MSCs become highly immunosuppressive upon stimulation by an inflammatory environment that leads to the production of nitric oxide (NO)^13^. On the other hand, MSCs that are unable to produce NO exhibit pro-inflammatory properties and promote immune responses^14^. The role of MSCs in haematopoiesis has been demonstrated by various studies. In certain culture conditions adherent stromal cells can support HSPCs^15^. Furthermore, MSCs can regulate haematopoiesis by producing various growth factors that are required for proliferation as well as differentiation of HSPCs expressing the CD34 surface marker.

Malaria parasites survive and replicate within erythrocytes by limiting immune responses^16^. On the other hand, immune components attempt to eradicate malaria parasites via CD4^+^ T cells^17^, γδ T cells^18^ and specific antibody production. In contrast, CD8^+^ T cells play a limited role during blood-stage malaria^19^, although these cells can lyse hepatocytes during the pre-erythrocytic stage of infection^20^. In order to proliferate, T cells require two signals from antigen-presenting cells (APCs). The first signal is generated by the ligation of the T cell receptor (TCR) complex with antigenic peptide bound with MHC molecules, and the second signal is in the form of activating co-stimulatory interactions with APCs. In addition to activating co-stimulatory interactions, the activity of T cells is also influenced by inhibitory co-stimulatory interactions with a variety of other cell types. Previously, it has been shown that programmed death-1 (PD-1), a member of the CD28 family of co-stimulatory molecules, plays a role in the outcome of malaria pathogenesis. Blockade or inhibition of PD-1 results in resistance against malaria infection^21^. PD-1 is induced in activated T, B and MSCs^22–25^, and interacts with its ligands programmed death-ligand 1 and 2 (PD-L1 and PD-L2) expressed by APCs. Engagement of PD-1 with its ligands causes exhaustion in T cells, resulting in blunting of T cell responses. A number of studies with various pathogens have reported that PD-1/PD-L interactions result in the inhibition of T and B cell proliferation^26–29^. Conversely, abrogation of such receptor/ligand interactions restores T cell functions and confers resistance against various pathogens. Previously, we have shown that inflammatory MSCs accumulate in the secondary lymphoid organs of mice during the progression of malaria infection, and that adoptive transfer of MSCs from infected mice to syngeneic animals protects against malaria challenge^30^. However, the mechanism underlying this protective function of MSCs remains incompletely understood. Two important events are crucial to impart protection against malaria infection. First, the generation of erythrocytes should be intact to avoid anaemia, which often arises due to the depletion of infected and uninfected RBCs during malaria parasite infection^31^. Second, there must be an appropriate induction and maintenance of protective cellular immune responses. CD34^+^ haemopoietic stem cells (HSC) with self-renewal capacity is required and sufficient for the differentiation of all blood cells^32^. The GATA family of transcription factors controls the development and homeostasis of various tissue systems, including blood. GATA-1, a zinc finger transcription factor, is essential for erythroid gene expression and maturation^33^. Furthermore, some reports have shown that both GATA-1 and GATA-2 are required for erythroid cell differentiation^34^. Moreover, mediator subunit Med1/TRAP220 is required for erythropoiesis just before the CFU-E stage as it acts as a co-activator for GATA- 1, deficiency of which severely affects CFU-E without affecting c-Kit-positive progenitor cells and BFU-E (burst forming units-erythrocytes) in embryos^35^. Therefore, the main focus of this study was to define the effect of MSC infusion on CD34^+^ haemopoietic cells, expression of GATA-1, and the proliferative and differentiation capacity of HSCs in the bone marrow. Further, we explored whether the expression of PD-1 was regulated and, in turn, the function of T cells was restored. Here we show that infusion of MSCs isolated from malaria-infected mice protects recipient animals from malaria infection, induces CD34+ cells in secondary lymphoid organs, restores erythropoiesis by upregulation of GATA-1, and restores T cells by lymphopoiesis. Further, T cell effector functions were improved by downregulation of PD-1 molecules. Taken together, these observations indicated that MSCs not only protect against malaria infection, but also improve adaptive immune responses.

## Results

### Fate of Sca-1^+^CD29^+^ cells after adoptive transfer to syngeneic animals

**Fig. 1:**
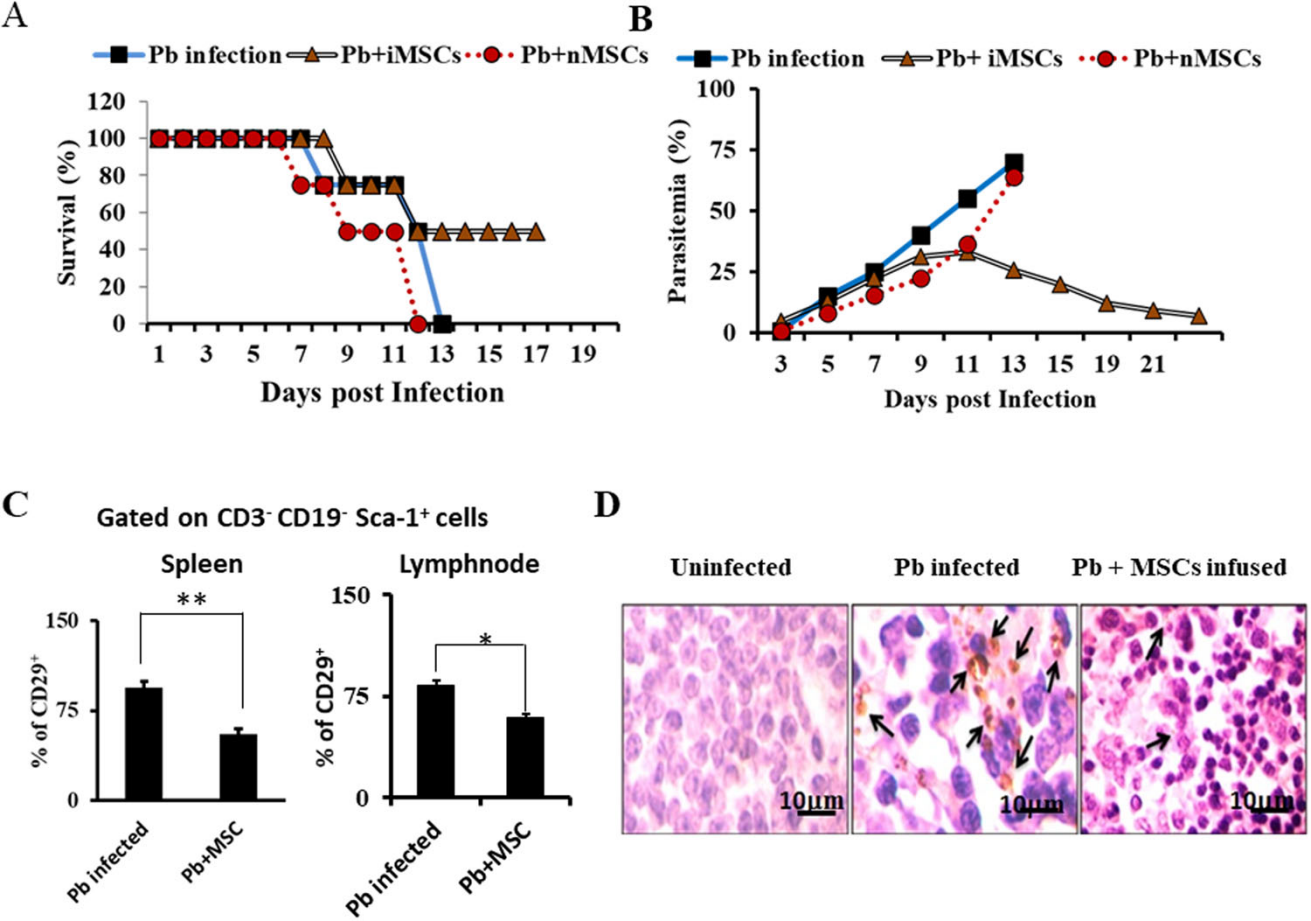
Mesenchymal stem cells protect from malaria infection. **A** Balb/c mice were infected with 5 × 10^5^ *Plasmodium berghei* (Pb) parasitized erythrocytes via intraperitoneal injection and were divided into three groups. One group of animals was injected with MSCs (5 × 10^6^ cells) from malaria-infected mice through the tail vein (iMSC, triangular, *n* = 9), the second group of mice was given MSCs from normal mice (nMSCs, Circle, *n* = 9) whereas the third group of mice received no cells (Square, *n* = 9). Blood collected from tails was used to prepare smears to determine parasitic load. **B** Survival of mice that received Sca-1^+^ cells from infected or normal mice versus control cells. Fifteen mice pooled from five independent experiments consisting of three mice in each group. **C** Cells were harvested from the spleen and lymph node, and stained with antibodies specific for MSCs. Cells were gated on the CD3^−^ CD19^−^ cell population and Sca-1^+^CD29^+^ cells in the spleen are shown. **D** Immunohistochemistry of CD29^+^ cells in the spleen of Balb/c mice (uninfected, infected with Pb, Pb-infected mice infused with MSC positive cells). Black arrows indicate the CD29- positive cells at 1000× original magnification. Statistical significance was determined by unpaired two-tailed Student’s *t*-test, *p < 0.05, **p < 0.01.

Previously, we have shown that MSCs play an important role in the outcome of malaria pathogenesis^30^. Here, we reaffirmed these results and performed more detailed analyses of the mechanisms involved. We isolated MSCs from malaria-infected mice (iMSCs) or control wild type mice (nMSCs) and adoptively transferred these cells to syngeneic animals, followed by infection with malaria parasites. As expected, we observed that infusion of MSCs dramatically lowered parasite growth (Fig. 1A) and increased survival of the animals (Fig. 1B), whereas MSCs from wild type mice did not show any change in parasitaemia and survival of infected mice, which is in agreement with our previous results^30^. Next, we intended to look at mechanisms adopted by these cells. Therefore, we determined the fate of these MSCs (Sca-1^+^CD29^+^) cells, after adoptive transfer to malaria parasite-infected mice. To do so, spleen and lymph nodes were harvested from malaria-infected mice infused with or without MSC on the 7th day post-infection. and stained with CD3, CD19 and Sca-1 and CD29 surface markers Surprisingly, animals that previously received MSCs exhibited profoundly reduced numbers of Sca-1^+^CD29^+^ cells than animals that did not receive MSCs both in spleen and lymph nodes (Fig. 1C). This observation was further confirmed by IHC of spleen sections with anti-CD29 antibody (Fig. 1D) by fixing a part of the spleen for immunohistochemistry (IHC staining). Consistent with the FACS data, we found reduced numbers of CD29-positive cells in MSC-infused compared with non-infused mice. These results indicate that infused MSC might be undergoing cell death and/or getting differentiated into some other lineages to help other cells to proliferate.

### Effects of MSCs on CD34^+^ haemopoietic cells

**Fig. 2:**
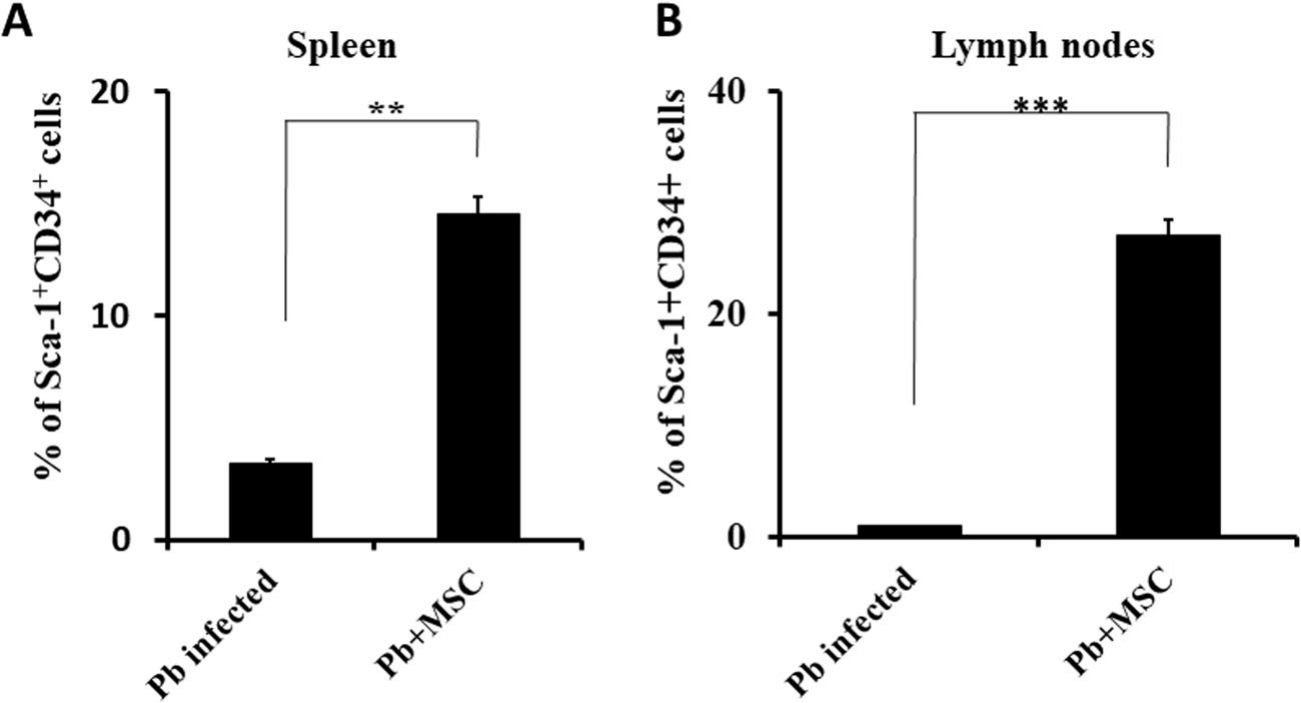
Infusion of MSCs upregulates CD34^+^ hematopoietic stem cells in various organs. Balb/c mice were infected with *Plasmodium berghei* and a group of mice received MSCs followed by Pb infection. These animals were sacrificed on day 7 post-infection and cells from various lymphoid organs were isolated and stained for CD3, CD19, Sca-1 and CD34 cell surface markers. The histogram represents the percentage of Sca-1^+^CD34^+^ cells in the spleen (**A**) and lymph node (**B**) (*n* = 9). Statistical significance was determined by unpaired two-tailed Student’s *t*-test; **p < 0.01 and ***p < 0.005.

From the preceding section, it is clear that MSCs assist in host protection against malaria infection. Next, we determined whether infused MSCs have any effect on hematopoietic cells by analysing the abundance of the CD34^+^ population, especially because mice infused with MSCs did not display anaemia. We harvested cells from spleen and lymph nodes from animals either infused with or without MSCs from malaria-infected mice. These cells were stained with anti-CD3, -CD19, -Sca-1 and -CD34 antibodies. The presence of CD34^+^ cells in these organs was determined by gating on CD3^−^CD19^−^Sca-1^+^ cells. Interestingly, we found a significant increase in Sca-1^+^CD34^+^ cells in the spleen (~5-fold) and lymph node (more than 20-fold) of MSC-infused as compared to uninfused mice (Fig. 2A, B). These observations suggested that MSCs influence the migration and differentiation of HSCs.

### Effects of MSCs on hematopoietic progenitor cell (HPC) proliferation and differentiation

**Fig. 3:**
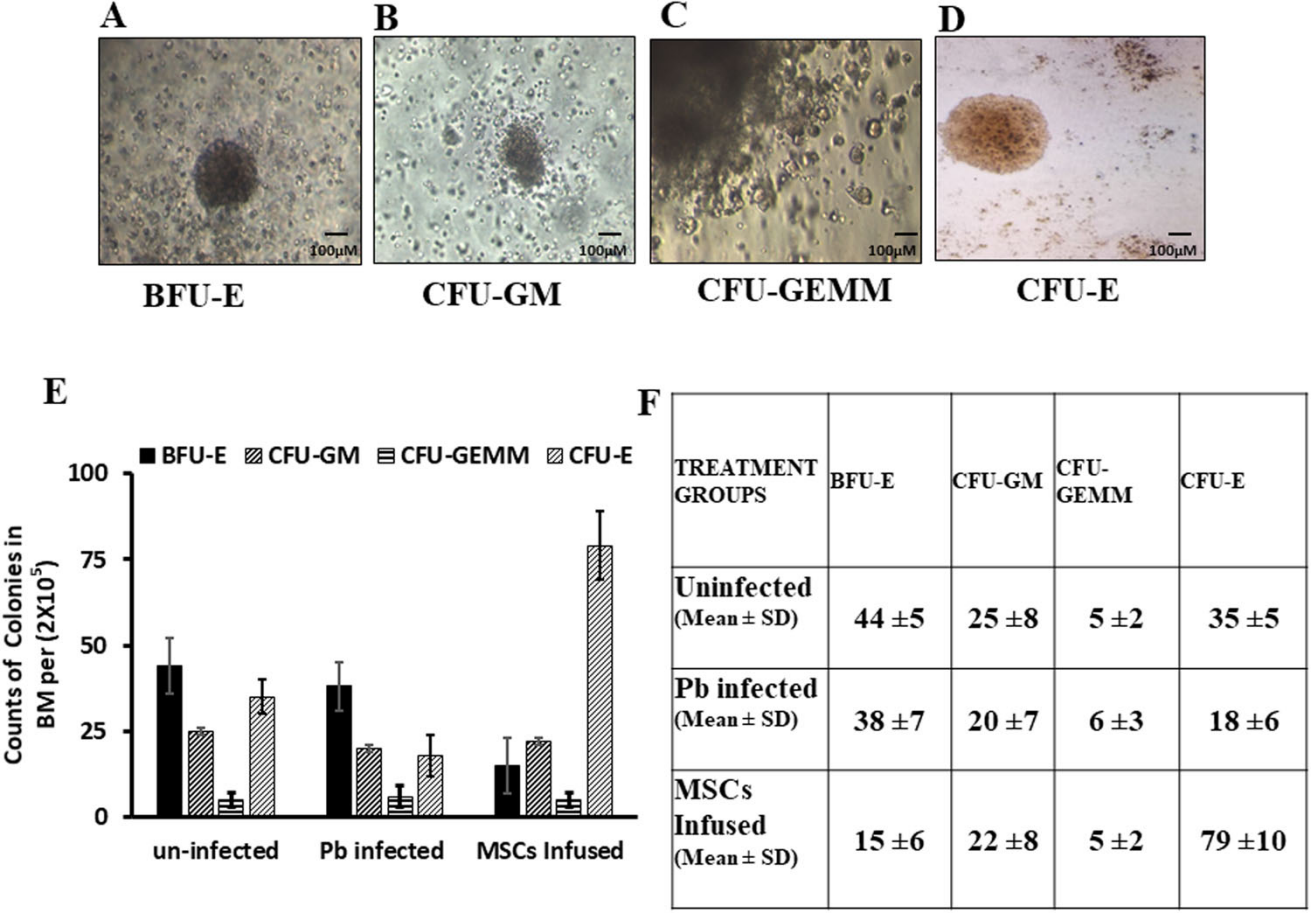
Infusion of MSCs induces proliferation and differentiation of haematopoietic progenitor cells. Bone marrow cells (2 × 10^5^) from malaria-infected mice with or without MSC infusion and uninfected control mice were cultured on semisolid (MethoCult) media. **A** BFU-E; **B** CFU-GM; **C** CFU-GEMM; **D** CFU-E. **E** Histogram representing the expansion of different colonies formed (CFU-GM, CFU-GEMM and BFU-E, *n* = 5). **F** Table showing the count of the number of colonies per plate. Data shown are mean value ± SD.

Next, we examined whether MSCs have any influence on the capacity of bone marrow HPCs to proliferate and differentiate into various other cell types. Bone marrow cells (2 × 10^5^) from malaria-infected mice with or without MSC infusion and from uninfected control mice were cultured on semisolid (MethoCult) media containing appropriate growth factors and supplements to support the growth of HPCs to proliferate and form colonies. These colonies can be either of the lymphoid lineage or myeloid lineage (granulocytic, monocytic, erythroid, and megakaryocytic lineages). The presence of CFU-E (later-stage erythroid progenitor cells) and BFU-E (primitive erythroid progenitor cells) represent the proliferative capacity of erythroid progenitors. These hematopoietic colonies were easily identified by their unique morphology and scored by microscopic examination (Fig. 3A–D). The in vitro colony-forming assays suggest that malaria infection leads to a reduction in the generation of CFU-GM, BFU-E, CFU-E and CFU-GEMM colonies compared to control mice (Fig. 3E). Interestingly, the number of BFU-E colonies was significantly higher in malaria-infected compared to mice previously infused with MSCs (Fig. 3F). We also observed that CFU-E colonies in malaria-infected mice were reduced, which significantly increased when MSCs were infused, suggesting that malaria infection causes an arrest in the differentiation of primitive HPCs (BFU-E) towards CFU-E (Fig. 3F).

This might be the reason why malaria infection results in dyserythropoiesis that ultimately leads to anaemia. Taken together, these data indicated that MSC infusion enhances proliferation and differentiation of bone marrow cells toward erythroid lineages, which correlates with increased host protection against infection by malaria parasites.

### Effects of MSCs on erythroid-specific transcription factors

**Fig. 4:**
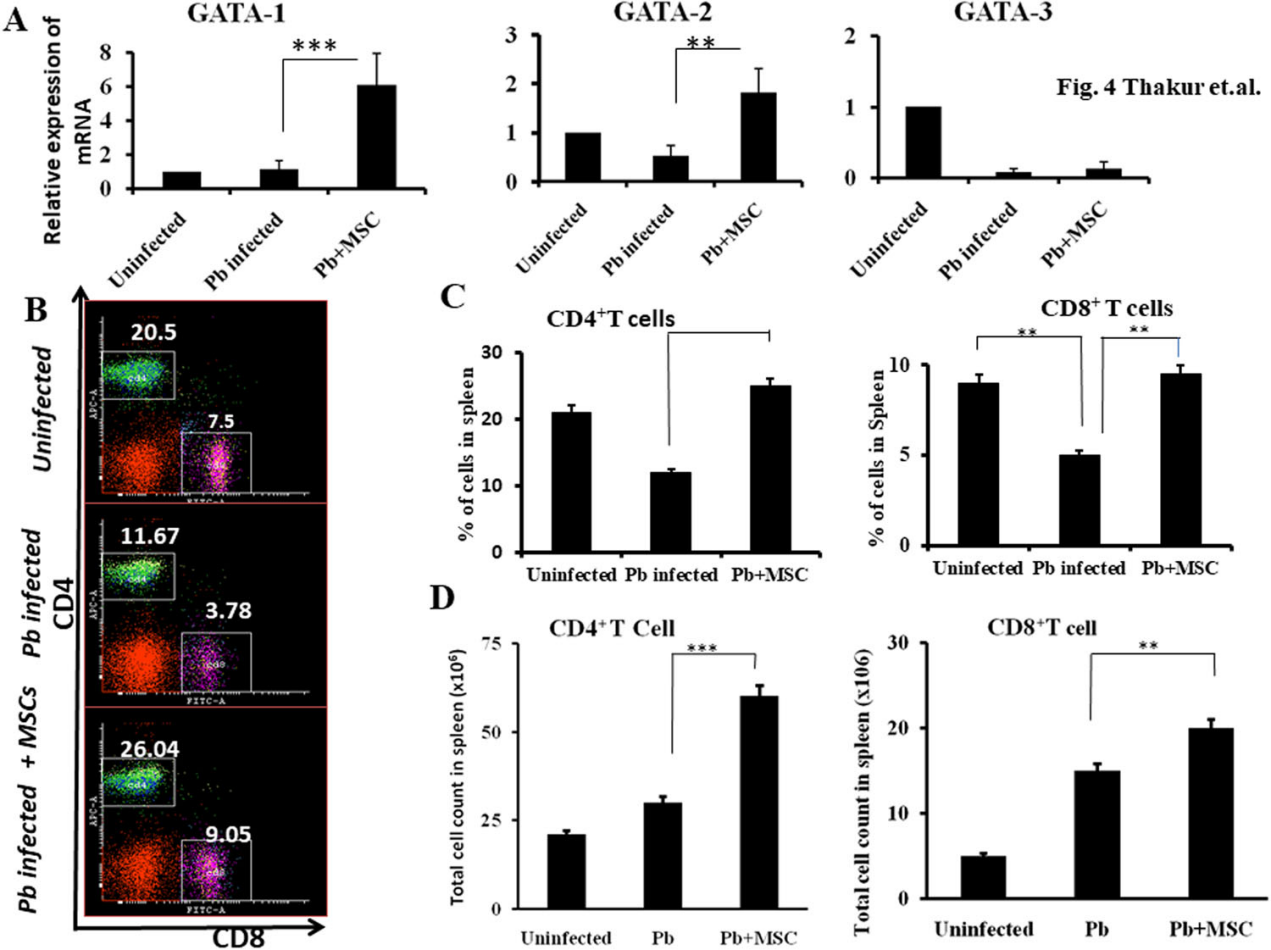
Infusion of MSCs induces erythroid-specific transcription factors and lymphopoiesis. Splenocytes were harvested at day 7 post-infection from Pb infected mice either infused with or without MSCs isolated from infected mice. RNA was used to perform relative quantification of the erythroid-specific transcription factors GATA-1, GATA-2 and GATA-3 (**A**). The number of CD4^+^ and CD8^+^ T cells from Pb infected mice either infused with or without MSCs was determined by staining with specific antibodies for cell surface markers and analysed by flow cytometry. **B**, **C** Percentage of CD4^+^ T cells and CD8^+^ T cells and **D** absolute number of CD4^+^ T cells and CD8^+^ T cells (*n* = 9). Statistical significance was determined by unpaired two-tailed Student’s *t*-test; **p < 0.01 and ***p < 0.005.

Hemozoin, parasite-derived polymerized heme produced during malaria infection, causes effective maturation of erythroids^36^. Next, we determined the expression of key GATA family transcription factors that are essential for erythropoiesis. We carried out relative expression analysis of these transcription factors by quantitative PCR. Mice that received MSCs exhibited at least a two-fold increase in the expression of GATA-1 as compared to the animals that did not receive MSCs. The low level of GATA-1 expression may be responsible for reduced RBC formation in malaria-infected animals. Although significant changes were observed in the expression of GATA-2, there was no significant expression of GATA-3 (Fig. 4A). These results further confirmed our conclusion that the infusion of MSCs elicits signals to assist in erythropoiesis, probably through increased CFU-E formation and reduced hemozoin content as observed in our earlier report^30^.

Lymphopoietic cells consisting of CD4^+^ and CD8^+^ T cells play critical roles in providing host protection in malaria infection. We found that the prevalence of both CD4^+^ and CD8^+^ T cells was decreased in mice during the progression of malaria disease (Fig. 4B, C). This might be due to the accumulation of other immune cells causing splenomegaly and unresponsiveness resulting in T cell death^37^. However, the absolute numbers of CD4^+^ and CD8^+^ T cells did not change significantly in malaria parasite infected as compared with control mice. On the other hand, prior infusion of MSCs prevented splenomegaly and accumulation of various cell types in the spleen, which in turn restored the abundance of CD4^+^ and CD8^+^ T lymphocytes (Fig. 4D). These results suggest that MSCs may have a causal relation with T lymphocyte abundance in malaria infection. We next examined whether MSCs have any influence on T cell proliferation as a means to rescue the functional properties these cells.

### Effects of MSCs on T cell proliferation

**Fig. 5:**
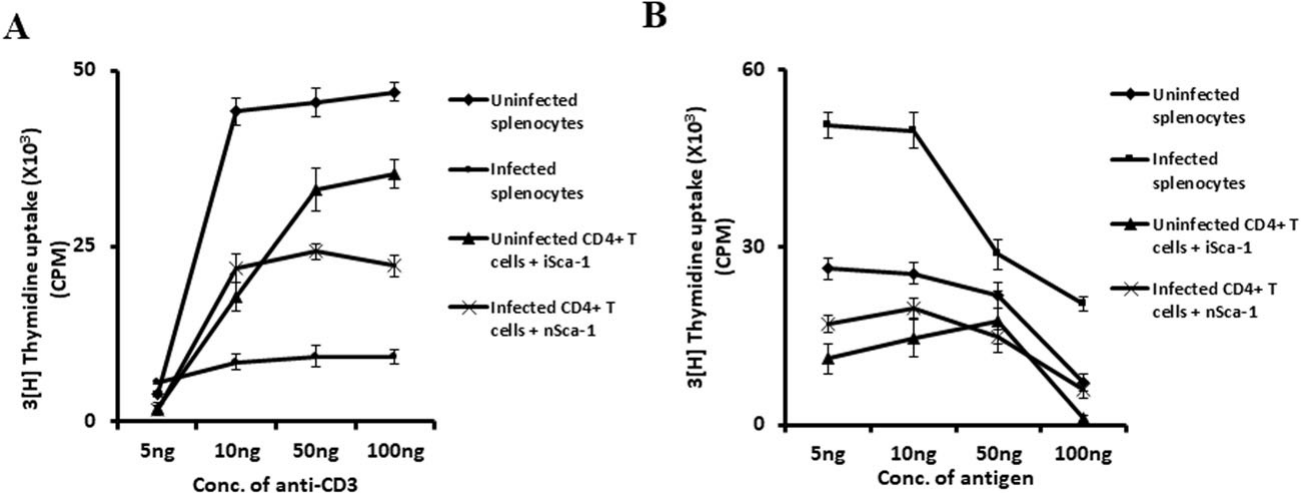
Enhanced T cell responses in the spleen of mice infected with malaria parasites are mediated by MSCs. Balb/c mice were infected with malaria parasites and on day 7 post-infection spleen CD4^+^ T cells were purified and co-cultured with lineage-depleted cells from splenocytes of infected or uninfected mice at a 2:1 ratio and activated with anti-CD3 antibodies (**A**) or malarial antigens (**B**). Proliferation was measured after 72 h by ^3^[H]-thymidine incorporation for 16 h by using scintillation beta-counter. Data shown are mean value ± SD.

Malaria infection results in the unresponsiveness of CD4^+^ T cells in vivo, due to exhaustion as evidenced by functional assays and expression of PD-1 molecules. To understand whether MSCs can restore CD4^+^ T cell proliferation, we examined T cell proliferation in response to polyclonal activation with anti-CD3 antibodies in the presence or absence of MSCs derived from infected or uninfected animals. We purified CD4^+^ T cells from the uninfected and infected animals and co-cultured them with MSCs derived from infected or uninfected animals, in the presence of lineage-depleted irradiated splenocytes as APCs. These cells were stimulated with various concentrations of anti-CD3 antibodies and the proliferative responses were measured through [^3^H]-thymidine uptake. Cultures that were supplemented with MSCs from infected animals exhibited better proliferative responses than cultures that received MSCs from uninfected mice (Fig. 5A). This observation suggested that T cell proliferation was enhanced by the presence of MSCs derived from infected mice, or the observed apparent differences might be due to T cell suppressive activities of MSCs from uninfected mice, whereas MSCs from infected mice were able to rescue T cell proliferation. Thus, T cell unresponsiveness can be restored by the presence of inflammatory MSCs. We further tested the malaria antigen-specific T cell responses. The T cells from malaria-infected mice showed elevated responses as compared to uninfected mice. The naive T cells failed to respond, even when co-cultured with lineage-depleted (inflammatory) cells from infected mice. MSCs, which are generally considered immunosuppressive in nature, upon co-culture with infected T cells, suppressed proliferation (Fig. 5B).

### Effect of MSCs on T cell PD-1 expression

**Fig. 6:**
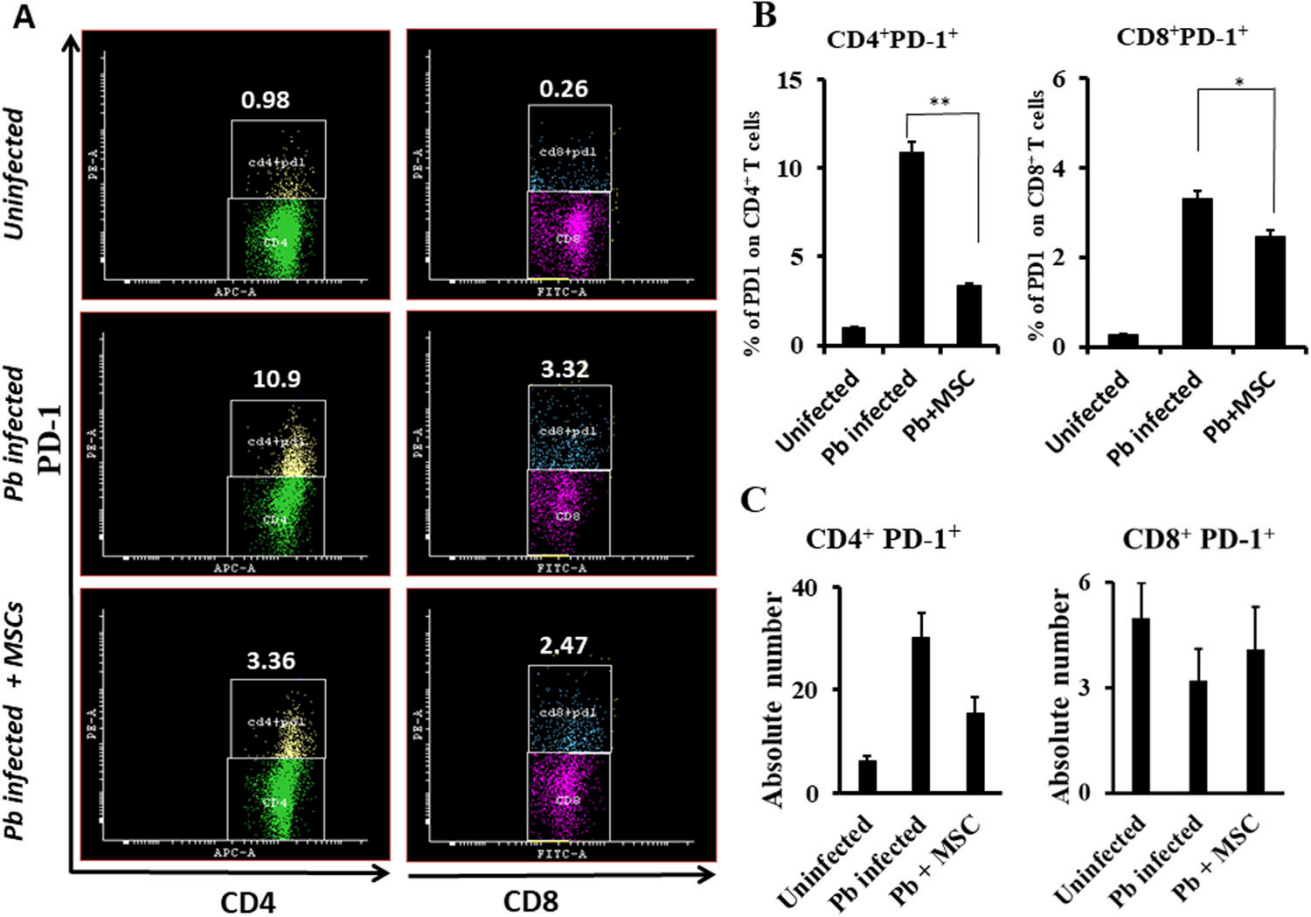
Effect of MSCs on the expression of PD-1 molecules by T cells. Spleen cells were harvested from *Plasmodium berghei* infected mice either infused with or without MSCs. Splenocytes were stained with PD-1-specific antibody along with anti-CD4 and -CD8 antibodies. Cells were gated on lymphocytes to determine CD4^+^ and CD8^+^ cells that were also positive for PD-1 expression. **A** Flow cytometry analysis shows the expression of PD-1 on CD4^+^ and CD8^+^ T cells in uninfected, and *Plasmodium berghei* infected mice either infused with or without MSCs (*n* = 9). **B** Percentage of CD4^+^ T cells and CD8^+^ T cells expressing PD-1 and **c** absolute number of CD4^+^ T cells and CD8^+^ T cells expressing PD-1. Statistical significance was determined by unpaired two-tailed Student’s *t*-test; **p < 0.01, *p < 0.05.

Next, we examined the expression of negative regulators that inhibit T cell responses, in an attempt to correlate this expression with poor effector T cell responses during malaria infection. We determined the expression of the immune checkpoint inhibitor PD-1 expressed on CD4^+^ and CD8^+^ T cells in malaria-infected mice. We found that both CD4^+^ and CD8^+^ T cells expressed significantly higher levels of PD-1 in infected than uninfected animals (Fig. 6A, B). Absolute numbers of PD-1-expressing CD4^+^ and CD8^+^ T cells among splenocytes of infected mice also showed a significant increase compared with the control group (Fig. 6C). Further, to understand the effects of MSC infusion on PD-1 expression, we isolated splenocytes cells from animals that were previously infused with MSCs followed by infection with malaria parasites, and determined the expression of PD-1 by FACS. Surprisingly, both CD4 and CD8 T cells from infused mice showed decreased expression of PD-1 compared to cells from uninfused but infected mice (Fig. 6B, C), suggesting that inhibition of the PD-1 pathway by the infusion of MSCs results in restoration of effector functions and, presumably, that recall responses result in improved effector immune responses and confer host protection

## Discussion

Previously, we have reported that Sca-1^+^ MSCs play an important role in host protection against malaria infection^30^. However, such host protective immune responses are greatly dependent on a variety of factors that include the dose of cells, route of administration and fate of cells after infusion. We standardized the infusion protocol to obtain an optimal protective immune response and then proceeded to study the mechanisms of host protection mediated by MSCs in malaria infection. The role of MSCs in the pathogenesis of infectious diseases, except for *Mycobacterium tuberculosis* infection^38^, has not been explored. *M. tuberculosis* infects MSCs and these cells in turn play a role in establishing dormancy of the mycobacterial organisms^39^. In contrast, malaria parasites do not infect MSCs, and these cells instead play a host protective role during infection^30^. These opposing roles of MSCs in different infections may be due to the involvement of different types of MSCs, with either inflammatory or immune-suppressive properties^13^. These diverse activities of MSCs are dictated by the microenvironment at the site of infection^12^. A similar report by Souza et al.^40^ also provides support for MSC-based cell therapy against cerebral malaria. Nevertheless, mechanisms of MSC-mediated host protection against malaria infection are unknown.

Dyserythropoiesis in malaria infection is a major cause of death and, hence, we explored whether MSCs influence erythropoiesis and the generation of anaemia. Since CD34^+^ HSCs differentiate to give rise to all blood cells, we examined numbers of CD34^+^ cells in animals that were infused with MSCs. We found increased CD34^+^ cells, which might contribute to the repair of malaria parasite-induced tissue injury. These findings are in agreement with the recent report by Hermida et al. showing that the expansion of CD34^+^ cells in the spleen has no relation with disease control during malaria infection^41^. We demonstrated that the infusion of MSCs restored haematopoiesis, which was dysregulated by malaria infection. This is indicated by increased numbers of CFU-E and reduced numbers of BFU-E in MSC-infused mice that are likely due to the differentiation of primitive erythroid colonies to late phase erythroid colonies. Moreover, cytokines such as IL-12 responsible for self-renewal and differentiation of multipotent progenitor cells are also induced, as reported previously^30^. Taken together, our results demonstrated that MSC infusion enhances haematopoiesis and replenishes the immune compartment.

Both CD4^+^ T and CD8^+^ T cells play a major role in protective immunity against malaria. The infection causes depletion of parasite-specific CD4^+^ T cells due to apoptosis, leading to impaired T cell-mediated immunity^42^. Our findings revealed that the infusion of MSCs from malaria-infected animals was able to rescue the proliferation of CD4^+^ T cells. Surprisingly, the number of CD4^+^ and CD8^+^ T cells was significantly increased in MSC-infused animals. The prevailing literature suggests that MSCs generally inhibit T cell activation due to the production of NO, IFN-γ and IDO^43^. Previously, we have shown that the MSCs induced during malaria infection neither produce NO nor exhibit immunosuppressive functions^30^, which is consistent with the proinflammatory subset of MSCs, and in agreement with the previous reports^14^. We further noticed that PD-1 expression is down regulated in animals that are infused with MSCs, once again consistent with the notion that these MSCs prevented immune suppression caused by malaria parasites. Taken together, these findings suggested that MSCs act through multiple signals that are capable of combatting malaria infection. MSCs promoted the reconstitution of erythroid and lymphoid cells, which could improve MSC engraftment. Stumpf and colleagues showed that Mediator subunit Med1/TRAP220 is required for erythropoiesis just before the CFU-E stage^35^. Since GATA-1 mainly affects early erythropoiesis and because MED1/TRAP220 interacts with GATA factors, it is likely that reduced expression of GATA-1 during malaria infection results in reduced erythropoiesis. GATA-1 is required for erythropoiesis and no other factors can compensate for its function in this process^44^. Nevertheless, the role of both GATA-1 and GATA-2 in erythropoiesis has been reported by Ohneda and Yamamoto^34^, suggesting a potential role for GATA-2 as well. As observed reduced formation of hemozoin might represent a further contributing factor towards improved erythropoiesis^30^^,45^. Thus, MSC- based therapies, unlike drugs that treat malaria, display therapeutic effects through several pathways. Our findings suggest that MSCs promote cellular immune responses that contribute to the establishment of protective immunity against malaria. Moreover, MSCs may be used as cell-based therapeutics for intervention in malaria that might cause sterile immunity. However, how paracrine mechanisms of MSCs act on the target tissues remains to be explored.

## Conclusions

These observations suggest that MSCs can protect mice from malaria infection by restoring CD34^+^ haemopoietic cells and CD4^+^ and CD8^+^ T lymphocytes, and by reducing the expression of negative co-stimulatory molecules on T lymphocytes. MSCs from infected mice exhibit enhanced immune stimulatory properties, as they can enhance T cell proliferation more profoundly than MSCs from uninfected mice. Studies to interrogate the signalling molecules engaged in restoring immune responses in malaria-infected mice by MSCs are underway.

## Materials and methods

### Animals and infection

Female Balb/c mice of 6 to 8 weeks of age were raised in the animal facility of the National Institute of Malaria Research, New Delhi (India) under a hygienic and pathogen-free environment. Cryopreserved Pb NK65 parasites (obtained from the parasite bank of the National Institute of Malaria Research, New Delhi, India) were passaged once in mice before use in experimental animals. Mice were infected with 5 × 10^5^ syngeneic parasitized erythrocytes (pRBCs) by intraperitoneal injection. The animals were used with the approval of the Institutional Animal Ethics Committee, in accordance with the guidelines of the Committee for the Purpose of Control and Supervision of Experiments on Animals (CPCSEA) approval no. IAEC/NIMR/2015/1.

### Determination of parasitemia

To determine the course of infection, blood smears were prepared from tail vein of animals at various time points as indicated in figure legends and stained with Jaswant Singh Bhattacharya (JSB) 1 and 2 stains. Parasitemia was determined by counting the percentage of infected cells per 5000 RBCs per slide.

### Antigen preparation

Blood was taken from *Plasmodium* infected animals with more than 20% parasitemia via cardiac puncture and pelleted at 700 × *g* for 5 min, and then washed with sterile 1×PBS. RBCs were lysed at 37 °C in a biochemical oxygen demand (BOD) incubator in 0.15% saponin for 30 min. After three additional washes the pellet was resuspended in PBS containing protease inhibitors followed by sonication.

### Cell preparation

Single-cell suspensions of splenocytes and lymph nodes (wherever described in figure legends) were prepared by smashing spleens of infected and control mice. RBCs were lysed with RBC lysis buffer (0.15 M NH_4_Cl, 10 mM KHCO_3_, 0.1 mM Na_2_EDTA). Cells were washed and then re-suspended in RPMI 1640 medium supplemented with 10% FBS (Hyclone, USA), 2 mM L-glutamine (Gibco-BRL, Life technologies, USA), and 100 IU/mL penicillin-streptomycin (Gibco-BRL).

### Flow cytometry

For flow cytometric analyses, spleen cells from mice were harvested on the 7th day post-infection, suspended in 50 μl of FACS buffer (PBS, 2% FCS) and surface stained at 4 °C with anti-mouse CD4 (GK1.5, Cat#17-0041-82) -CD8 (53-6.7 Cat#11-0081-82), -CD19 (1D3, Cat#17 0193-82), -CD29 (HM-Bta1-1 Cat#12-0291-82), -CD3 (2C11 Cat#17-0032-82), -Sca-1 (Ly-6A/E Cta#11-5981-82) PD-1 (MIH4 Cat#12-9969-42) were purchased from e-Biosciences and -CD34 (RAM34 Cat#551387 BD Biosciences) Stained cells were acquired with a FACS Fortessa (BD Biosciences) and analysed by Flow Jo (Tree star) or cyflogic (CyFlo Ltd, Finland) software.

### Adoptive transfers of MSCs

For adoptive transfer experiments, MSCs were purified by depletion of lineage-differentiated cells such as T lymphocytes, B lymphocytes, macrophages, and DCs (using micro-beads from Miltenyi-Biotec). Lineage-depleted cells (5 × 10^6^) were adoptively transferred into syngeneic animals intravenously (i.v.) through the tail vein, followed by injection of malaria parasites intra-peritoneally (i.p.).

### CFC (colony-forming cell/unit) assay

Balb/c mice of all three treatment groups (control, Pb-infected, and MSCs infused into Pb-infected) were employed. At the 7th day post-infection, mice were sacrificed and BM (bone marrow) cells from the femur were flushed using HBSS. BM cells were taken out and washed in HBSS by centrifugation at 1000 rpm for 10 min. Pellets were resuspended in 0.5 ml MEM media containing 2% serum. Crude BM cells (2 × 10^5^ cells) containing HSCs were cultured on semi-solid MethoCult media (Stem Cell Technologies, Canada) to observe various in vitro colony-forming units of the hematopoietic lineage. Cells were cultured in 35 mm petri-dishes in triplicates. Colonies formed after 10–12 days of incubation were counted under ×10 magnification of a light microscope. Different colonies formed (CFU-GM, CFU-GEMM and BFU-E) were counted for all treatment groups.

### Real-time PCR

Total RNA was isolated from splenocytes using RNeasy mini kit (Qiagen, USA). Genomic DNA was removed using the RNase-free DNase set for DNA digestion during RNA purification. First-strand cDNA synthesis was performed using a Superscript cDNA synthesis kit (Invitrogen). Levels of mRNA of the gene of interest were quantified by real-time PCR (Roche 480 Lightcycler) using SYBR Green Master Mix (Applied Biosystems). Thermocycling included an initial incubation at 95 °C for 5 min, then 95 °C for 10 s, followed by 60 °C for 10 s, then 72 °C for 10 s. Melting curve temp was followed by 95 °C for 5 s, and 65 °C for 1 min for a total of 45 cycles. The total amount of mRNA was normalized across samples according to endogenous actin mRNA. Primer sequences were as follows: beta-actin-Forward 5′-GATCATGTTTGAGACCTTCAACACC-3′ beta-actin Reverse 5′-CGTGAGGGAGAGCATAGCCC-3′,GATA1-Forward 5′-GGAGGAATGCCAGCCGAGAT-3′GATA1-Reverse 5′-TGCAGTGCCCAGTGCCAAGC-3′GATA2-Forward 5′-TCAGACGACAACCACCACCTTA-3′ GATA2 Reverse 5′-ATTTGCTGGACATCTTCC GATT-3′ GATA3 Forward 5′-TTATCAAGCCCAAGCGAAGG-3′ GATA3 Reverse 5′-CATTAGCGTTCCTCCTCCAGAG-3′.

### Histological analyses

Spleens from the infected and control wild type mice were analysed for histopathology. Sections of spleen tissue were cut into 5 μm sections by microtome, and probed with anti- CD29 antibodies (eBioscience, Inc USA), and analysed by IHC method described elsewhere^46^. All histology slides were examined in a double-blinded manner.

### [^3^H]-Thymidine incorporation assay of splenocytes

Spleens were harvested from malaria parasite-infected mice and control animals and single-cell suspensions were made using frosted slides as described^47^. RBCs were lysed with RBC lysis buffer and washed with complete RPMI 1640. Splenocytes were counted, plated at 5 × 10^5^ cells/well in 96-well plates and stimulated with soluble anti-CD3 antibody. Cultures were supplemented with different concentrations of malarial antigens or soluble anti-CD3 antibodies as indicated in the figure. The CD4^+^ T cells from infected or uninfected animals were purified and co-cultured with lineage-depleted cells from splenocytes of infected or uninfected mice at a 2:1 ratio and vice versa. These cells were cultured at 37° C in a CO_2_ incubator for 72 h and then pulsed with tritiated thymidine (^3^H-TdR, 0.5 µCi per well; Amersham Biosciences, UK) before measuring the incorporation of ^3^H-TdR by a cell harvester and liquid scintillation counter, 16 h later (Wallac-Trilux, Perkin Elmer, UK).

### Statistical analysis

All statistical analysis was performed in MS excel 2003. Unpaired two-tailed Student’s *t*-test was used as indicated in figures and differences were considered significant when *p* ≤ 0.05.
